# A Tale of Two Oxidation States: Bacterial Colonization of Arsenic-Rich Environments

**DOI:** 10.1371/journal.pgen.0030053

**Published:** 2007-04-13

**Authors:** Daniel Muller, Claudine Médigue, Sandrine Koechler, Valérie Barbe, Mohamed Barakat, Emmanuel Talla, Violaine Bonnefoy, Evelyne Krin, Florence Arsène-Ploetze, Christine Carapito, Michael Chandler, Benoît Cournoyer, Stéphane Cruveiller, Caroline Dossat, Simon Duval, Michael Heymann, Emmanuelle Leize, Aurélie Lieutaud, Didier Lièvremont, Yuko Makita, Sophie Mangenot, Wolfgang Nitschke, Philippe Ortet, Nicolas Perdrial, Barbara Schoepp, Patricia Siguier, Diliana D Simeonova, Zoé Rouy, Béatrice Segurens, Evelyne Turlin, David Vallenet, Alain Van Dorsselaer, Stéphanie Weiss, Jean Weissenbach, Marie-Claire Lett, Antoine Danchin, Philippe N Bertin

**Affiliations:** 1 Génétique Moléculaire, Génomique et Microbiologie, UMR7156 CNRS and Université Louis Pasteur, Strasbourg, France; 2 Génoscope, UMR8030 CNRS, Evry Cedex, France; 3 Laboratoire d'Écologie Microbienne de la Rhizosphère et d'Environnements Extrêmes, UMR6191 CNRS, CEA and Université Aix-Marseille II, Saint-Paul-lez-Durance, France; 4 Laboratoire de Chimie Bactérienne, UPR9043 CNRS, Institut de Biologie Structurale et Microbiologie, Marseille, France; 5 Génétique des Génomes Bactériens, URA2171, Institut Pasteur, Paris, France; 6 Laboratoire de Spectrométrie de Masse Bio-Organique, Institut Pluridisciplinaire Hubert Curien, UMR7178 CNRS and Université Louis Pasteur, Strasbourg, France; 7 Laboratoire de Microbiologie et Génétique Moléculaires, UMR5100 CNRS, Toulouse, France; 8 Ecologie Microbienne, UMR5557 CNRS and Université Claude Bernard–Lyon 1, Villeurbanne, France; 9 Laboratoire de Bioénergétique et Ingénierie des Protéines, UPR9036 CNRS, Institut de Biologie Structurale et Microbiologie, Marseille, France; 10 Centre de Géochimie de la Surface, UMR7517 CNRS and Université Louis Pasteur, Strasbourg, France; Department of Energy Joint Genome Institute, United States of America

## Abstract

Microbial biotransformations have a major impact on contamination by toxic elements, which threatens public health in developing and industrial countries. Finding a means of preserving natural environments—including ground and surface waters—from arsenic constitutes a major challenge facing modern society. Although this metalloid is ubiquitous on Earth, thus far no bacterium thriving in arsenic-contaminated environments has been fully characterized. In-depth exploration of the genome of the β-proteobacterium Herminiimonas arsenicoxydans with regard to physiology, genetics, and proteomics, revealed that it possesses heretofore unsuspected mechanisms for coping with arsenic. Aside from multiple biochemical processes such as arsenic oxidation, reduction, and efflux, H. arsenicoxydans also exhibits positive chemotaxis and motility towards arsenic and metalloid scavenging by exopolysaccharides. These observations demonstrate the existence of a novel strategy to efficiently colonize arsenic-rich environments, which extends beyond oxidoreduction reactions. Such a microbial mechanism of detoxification, which is possibly exploitable for bioremediation applications of contaminated sites, may have played a crucial role in the occupation of ancient ecological niches on earth.

## Author Summary

Microorganisms play a crucial role in nutrient biogeochemical cycles. Arsenic is found throughout the environment from both natural and anthropogenic sources. Its inorganic forms are highly toxic and impair the physiology of most higher organisms. Arsenic contamination of groundwater supplies is giving rise to increasingly severe human health problems in both developing and industrial countries. In the present work, we investigated the metabolism of this metalloid in *Herminiimonas arsenicoxydans,* a representative organism of a novel bacterial genus widespread in aquatic environments. Examination of the genome sequence and experimental evidence revealed that it is remarkably capable of coping with arsenic. Our observations support the existence of multiple strategies allowing arsenic-metabolizing microbes to efficiently colonize toxic environments. In particular, arsenic oxidation and scavenging may have played a crucial role in the development of early stages of life on Earth. Such mechanisms may one day be exploited as part of a potential bioremediation strategy in toxic environments.

## Introduction

Although arsenic is most notorious as a poison threatening human health [1], recent studies suggest that arsenic species may have been involved in the ancestral taming of energy and played a crucial role in early stages in the development of life on Earth [2,3]. Further speculations involve this metalloid in the colonization of extraterrestrial environments containing high arsenic levels [4,5]. Presently, arsenic contamination of drinking water constitutes an important public health problem in numerous countries throughout the world [6]. Elevated concentrations typically derive from the weathering of arsenic-bearing minerals or from geothermal sources; lower amounts are of anthropogenic origin, e.g., smelting and mining industries.

Microorganisms are known to influence arsenic geochemistry by their metabolism, i.e., reduction, oxidation, and methylation [7,8], affecting both the speciation and the toxicity of this element. Arsenate (As[V]) is less toxic than arsenite (As[III]), but, paradoxically, resistance to As[V] requires its reduction to As[III], which will be extruded. On the other hand, arsenite oxidation, which was primarily thought to constitute a detoxification mechanism [9], may serve as an energy source in chemilithotrophic microorganisms [10]. Bacteria metabolizing toxic elements represent therefore an attractive tool to restore contaminated sites. In this respect, H. arsenicoxydans strain ULPAs1, which oxidizes As[III] into its less toxic and more easily immobilized form As[V], has been proposed for use in the first steps of arsenic bioremediation [11].

This heterotrophic microorganism, formerly called Caenibacter arsenoxydans ULPAs1, was isolated from the activated sludge of an industrial water treatment plant contaminated with heavy metals such as arsenic, lead, copper, and silver [12]. In the Burkholderiales order, its nearest phylogenetic relatives are members of the Oxalobacteraceae/Burkholderiaceae families, which contain several natural isolates with important biotechnological properties. For example, bacteria of the *Paucimonas* [13] and *Collimonas* [14] genera are known for their polyhydroxybutyrate depolymerase and chitinase activity, respectively. H. arsenicoxydans is a representative strain of a new genus comprising bacteria isolated from various aquatic environments, including contaminated, mineral and drinking water [12,15,16]. To gain further insight into the mechanisms that permit the microbial colonization of arsenic-rich environments, we investigated the physiology of H. arsenicoxydans by genetic and functional approaches*.* The results reported here, associated with descriptive and comparative genomics data, emphasize the metabolic versatility of this strain with regard to arsenic and the ability of microorganisms to restore liveable conditions within their ecological niche.

## Results

### General Genome Features

**Figure 1:**
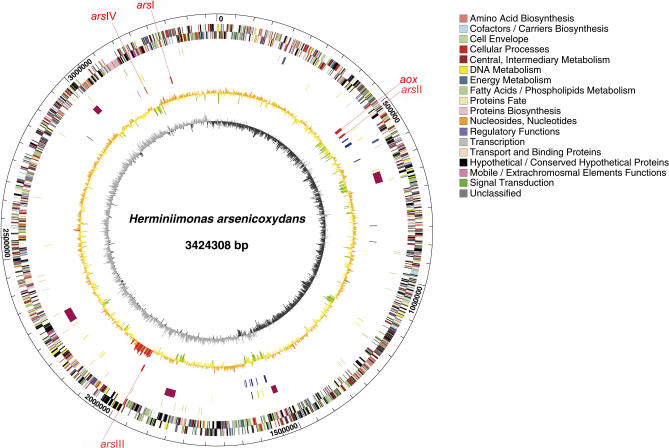
Circular Representation of H. arsenicoxydans Genome Circles display (from the outside inward): (rings 1 and 2) predicted CDSs transcribed in a clockwise/counterclockwise direction, (ring 3) tRNA (green) and rRNA (violetblue), (ring 4) ISs (yellow) and prophagic regions (violetbrown), (ring 5) clusters of genes involved in arsenic (red; numbered as shown in Figure 4)/metal resistance (dark blue), (ring 6) GC deviation (GC_window_ − average GC of genome, using a 1kb window); (7) GC skew (using a 1kb window). The high GC-rich island described in Figure 2 is shown in red.

The H. arsenicoxydans genome consists of a single circular chromosome of 3,424,307 bp (Figure 1) with a total of 3,333 coding sequences (CDSs), among which 38% are of unknown function (Table 1). Surprisingly, any attempt to identify extrachromosomal elements in this strain by DNA sequencing, pulse-field electrophoresis, or plasmid purification was unsuccessful (unpublished data). This suggests that, unlike many microbes isolated from natural or anthropized environments, H. arsenicoxydans contains neither a second chromosome nor a (mega)plasmid. In bacteria, mobile genetic elements are known to play a major role in the acquisition of genes involved in adaptation to environmental stresses [17]. In line with this observation and in contrast to the situation in related microorganisms such as Ralstonia metallidurans [18], only a small number of complete or partial insertion sequences (IS) were identified in the genome of H. arsenicoxydans (Table S1). These IS elements represent 0.65% of the genome and belong to several families; i.e., IS3, IS30, and IS110*. H. arsenicoxydans* also contains more complex transposons or transposon remnants, but, unlike those identified in biomining strains used for metal recovery from gold-bearing arsenopyrite ores [19], they do not convincingly harbor arsenic-resistance determinants. Interestingly, all the complete ISs are inserted with their transposase genes in a clockwise orientation with respect to the orientation of the replication fork, suggesting some sort of interference between replication and IS stability.

**Table 1:**
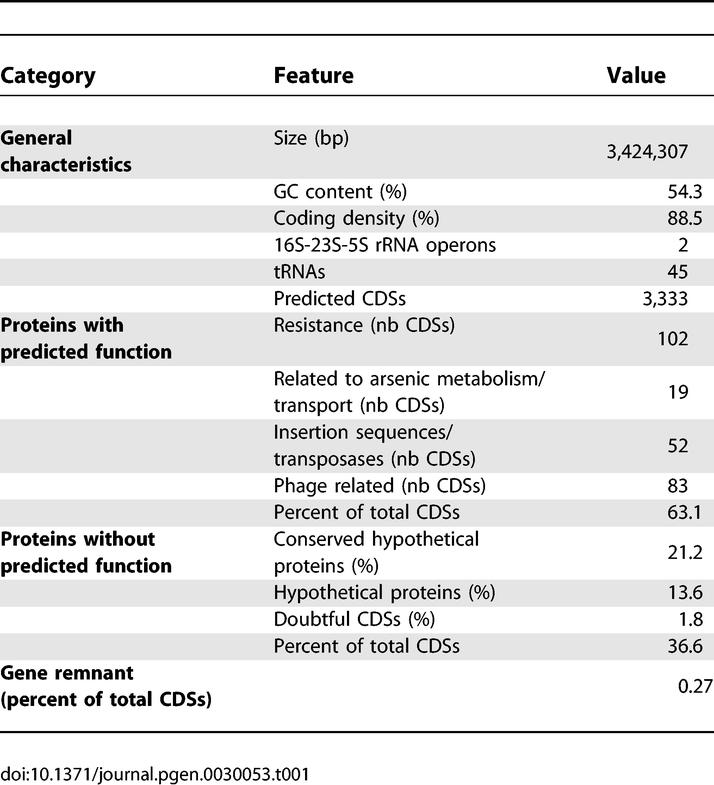
General Features of the H. arsenicoxydans Genome

**Figure 2:**
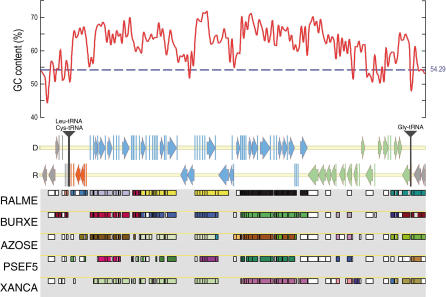
Detailed View of a GC-Rich Island The chromosomal segment extends between positions 1.97 and 2.07 Mb on the H. arsenicoxydans chromosome. Frames display (from top to bottom): (1) %GC along this island; (2) annotated CDSs on the direct (D) and reverse (R) strand: *ars*III gene cluster (six genes in red arrows), part of the *clc* element of plasmid (or phage) origin, initially described in Pseudomonas sp. strain B13 [22] (64 genes in light blue arrows), and phage-related function (DNA repair, integrase) associated with metabolic capabilities, such as formaldelyde oxidation (17 genes in light green arrows), small genes are represented by a line; (3) synteny maps, calculated on a set of selected genomes (RALME, Ralstonia metallidurans CH34; BURXE, Burkholderia xenovorans LB400; AZOSE, Azoarcus sp. EbN1; PSEF5, Pseudomonas fluorescens Pf-5; and XANAC, Xanthomonas campestris 85–10). A line contains the similarity results between H. arsenicoxydans and one given genome. A rectangle represents a putative ortholog between one CDS of the compared genome and the CDS of the H. arsenicoxydans genome opposite. When, for several CDSs colocalized on the H. arsenicoxydans genome, several colocalized orthologs have been identified in the compared genome, the rectangles will be of the same color. Otherwise, the rectangle is white. A group of rectangles of the same color therefore indicates the existence of a synteny between H. arsenicoxydans and the compared genome, using a gap parameters of five genes maximum [63]. Details on correspondences between genes in the synteny (Table S2) show that the light blue section of this island in H. arsenicoxydans is also found at the same chromosomal location in the compared genomes.

The overall GC content of the H. arsenicoxydans genome is 54.3 % but seven regions exhibit a lower content and one region a higher content than the average (Figure 1). The presence of ISs was recorded in six of the low-GC modules. Remarkably**,** the region with the highest GC content (63%), harbors several CDSs (coding sequences) identified as homologs of phage and/or plasmid-like genes coding for proteins involved in chromosome partitioning, DNA topoisomerase, DNA helicase, and DNA recombination and repair (Table S2). This region, which covers ∼90 kb in the H. arsenicoxydans genome, is bordered by two tRNA genes at one end and one tRNA gene at the other end, and is flanked on one side by an integrase gene (Figure 2), suggesting a probable acquisition by an RNA-mediated horizontal gene transfer [20]. In terms of similarity, this island is clearly made of three main parts (Figure 2). The first one contains an arsenic-resistance cluster, also found in *R. metallidurans,* and, to a lesser extent, in Azoarcus sp. and Pseudomonas fluorescens. The second part of this region harbors a set of genes highly similar to part of the *clc* genomic island originally discovered in Pseudomonas sp. strain B13, which is known for its ability to degrade chloroaromatic compounds [21]. The *clc* element is almost 100% identical over the whole length (102 kb) to a chromosomal region in the chlorobiphenyl-degrading bacterium Burkolderia xenovorans LB400 [22]. Interestingly, a similar region of conserved synteny was observed in various proteobacteria such as R. metallidurans, *P. fluorescens, Xanthomonas campestris,* and Azoarcus sp., but none of the catabolic properties described in the *clc* element, mainly the *clc* and the *amn* operons (allowing 3-chlorobenzoate and 2-aminophenol degradation, respectively), were found in this part of the H. arsenicoxydans island (Table S2). Specific metabolic capabilities are found in the third part of this region, especially glutathione-dependent and -independent enzymatic activities involved in formaldelyde oxidation. Finally, several resistance genes found in the *clc* element of the compared genomes (e.g., mercuric resistance in R. metallidurans or ultraviolet-light resistance in P. fluorescens, Table S2) support a role for this genomic island in the adaptive response to stressful environmental conditions.

### Carbon and Energy Metabolism

Many bacterial strains of the *Burkholderiales* order are able to flourish in diverse ecological niches and grow on various carbon sources. Surprisingly, H. arsenicoxydans can metabolize only a limited number of organic acids such as lactate, oxalate, succinate, and acetate; this is consistent with the presence of the corresponding functions on the chromosome and the absence of carbohydrate transporter genes such as those found in the phosphostranferase system (Table S3). In addition, the use of amino acids as a sole carbon and nitrogen source is supported by the ability of the strain to grow on tryptone and the presence in its genome of multiple operons coding for amino acid transport systems**.** In contrast, none of the pathways enabling carbon fixation from CO_2_ (i.e., genes coding for ribulose 1,5-biphosphate carboxylase/oxygenase and those involved in the Calvin cycle) are present or complete, in conformity with the chemoheterotroph metabolism of *H. arsenicoxydans.*

Genes involved in the biosynthesis or degradation of glycogen were not identified in the genome of H. arsenicoxydans. In contrast, the presence of the *phbA-phbB* gene cluster, which encodes a β-ketothiolase and an acetoacetyl-coenzyme A reductase, and *phbC,* coding for a poly-beta-hydroxybutyrate polymerase, is consistent with the accumulation in H. arsenicoxydans of poly-beta-hydroxybutyrate as an intracellular energy storage material (unpublished data), as recently demonstrated in Ralstonia eutropha [23]. Moreover, the genome contains all the genes encoding the inorganic phosphate transport and the phosphate-specific transport systems [24,25], as well as genes possibly involved in the synthesis of high-energy polyphosphate granules (Table S3), which may constitute an additional means of energy storage for *H. arsenicoxydans.*

The diversity of electron transfer mechanisms is of prime importance in the management of energy in ecosystems subjected to frequent fluctuations in their oxygen content, such as water treatment plants. Genomic data analyses suggest that H. arsenicoxydans can accommodate a wider range of oxygen concentrations than was initially anticipated [12,26]. Indeed, the *H. arsenicoxydans* genome harbors multiple respiratory pathways, permitting microorganisms to grow under aerobic, microaerobic, and anoxic conditions (Table S3). Reducing equivalents derived from organic compounds can enter energy-conserving electron transfer chains via a succinate dehydrogenase and three distinct formate dehydrogenases, none containing selenocysteine. Possible inorganic electron donors are reduced sulfur compounds (with the notable exceptions of sulphite and dimethyl sulphite) and As[III]. In contrast*,* no hydrogenase-encoding genes have been detected, suggesting that the strain may not gain energy from the oxidation of H_2_ to protons. At the oxidizing end of bioenergetic electron transfer chains, five terminal oxidases might be operative (Table S3). The two *caa*_3_ cytochrome oxidases usually operate under high oxygen tension while *bo*_3_, *cbb*_3,_ and *bd* oxidases are more specific to low-oxygen conditions [27]. All enzymes involved in anaerobic respiration via denitrification have been identified in the genome, i.e., nitrate, nitrite, nitrous oxide, and nitric oxide reductases. A β-proteobacterial cytochrome *bc*_1_-complex serves as a coupling site in many of these energy-conserving chains [28].

The genome was explored to identify genes coding for cytochrome proteins possibly facilitating electron transfer between the Aox system and the *bc1* complex and *cbb3* cytochrome oxidase. The consensus sequence for the cytochrome *c* center is Cys-x-x-Cys-His, in which the histidine residue is one of the two axial ligands of the heme iron. Among the 56 putative heme-binding proteins we identified, Hear0476 was a particularly attractive candidate, because this protein was not predicted as a subunit of a cytochrome containing system, its coding gene was located immediately downstream of the *aoxABC* operon, and its expression was induced in the presence of arsenic (Table 2). This putative protein belongs to the *c*552 family and was named AoxD. Such a cytochrome has been shown to interact with the terminal cytochrome *cbb3* in Helicobacter pylori [29], to play the role of electron carrier to the *bc1* complex in ammonia-oxidizing bacteria [30], and to coprecipitate with As[III] oxidase protein in Alcaligenes faecalis [9]. We therefore propose that AoxD represents the electron transfer link between AoxAB proteins and the *ccb3* cytochrome oxidase and *bc1* complex in H. arsenicoxydans.

**Table 2:**
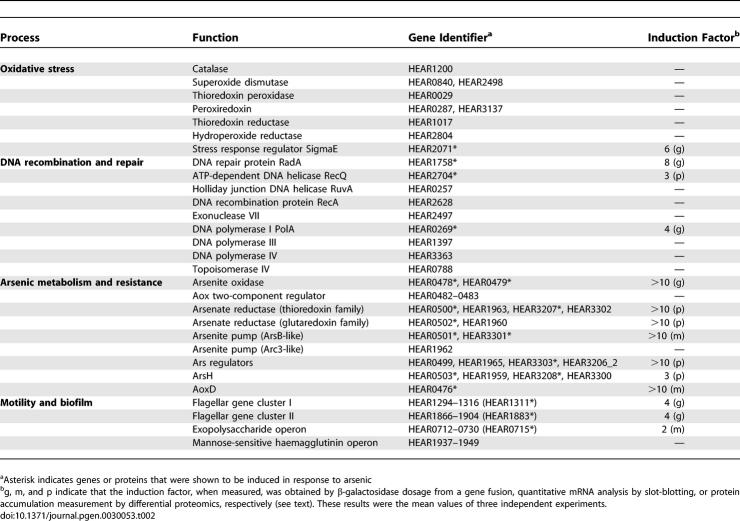
Genetic Determinants of Arsenic-Related Stress Adaptation

Finally, any attempt to cultivate H. arsenicoxydans ULPAs1 with As[III] as an electron donor source was unsuccessful; this organism requires an organic compound as an energy source. Moreover, neither selenate reductase nor respiratory arsenate reductase was identified in the genome. In Shewanella sp., this latter enzyme is encoded by the *arrAB* locus and allows anaerobic respiration with As[V] as a terminal electron acceptor [31].

### Arsenic Stress and Metal Resistance

H. arsenicoxydans is not only resistant to arsenic but also to various heavy metals such as cadmium and zinc (Table S4). This observation is consistent with the presence in its genome of multiple metal-efflux operons (Figure S1), e.g., three cobalt-zinc-cadmium *czc* operons. However, except for arsenic, the resistance levels to toxic metals were much lower than those measured in the metallophilic R. metallidurans (Table S4), which contains multiple plasmid-encoded genes [18], suggesting a specific physiological adaptation of H. arsenicoxydans towards the arsenic.

Exposure to arsenic results in various biological effects, including DNA damage and oxidative stress [32,33]. H. arsenicoxydans exhibits both positive oxidase and catalase activities [12], in agreement with the presence of one catalase and two superoxide dismutase–encoding genes (Table 2). The genome also encodes at least one thioredoxin peroxidase, one peroxiredoxin, one thioredoxin reductase, and one hydroperoxide reductase. Moreover, genes coding for bacterioferritin and bacterioferritin comigratory protein, known to protect cells against toxic hydroxyl radicals resulting from iron overload, could also play a role in the adaptive response to oxidative stress [34]. In addition, the partial screening of a Tn*5-lacZ* mutant library demonstrated an induction of several genes involved in DNA recombination and repair, e.g., *radA* and *polA*, in the presence of arsenic (Table 2). Their inactivation in H. arsenicoxydans led to an important loss of viability following UV exposure, which was further decreased by arsenic (Figure S2). This suggests that the metalloid exerts a significant effect on DNA integrity.

**Figure 3:**
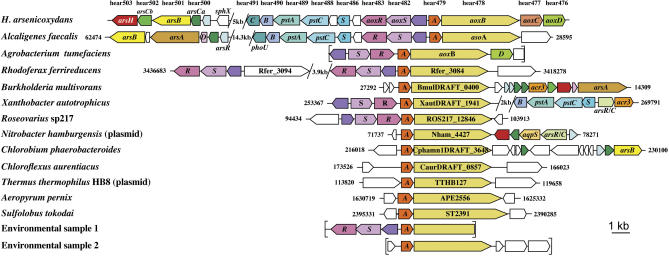
Organization of the *aox* Gene Cluster in H. arsenicoxydans and Various Arsenic-Metabolizing Microorganisms The *aoxAB* operon is close to arsenic-resistance genes in H. arsenicoxydans, *A. faecalis, X. autotrophicus, N. hamburgensis,* and *C. phaeobacteroides.* In the first three bacteria, these genes are associated with an *aoxRS* two-component regulatory system. In *H. arsenicoxydans,* the CDS number of *aoxABCD, aoxRS,* and *arsRCBCH* are *hear*0479–0476, *hear*0483–0482, and *hear*0499–0503, respectively. Sequence information of other genes was obtained from GenBank database and their localization on the chromosome or the plasmid is given by nucleotide numbering. The following bacterial genomes were used: *Alcaligenes faecalis*, Agrobacterium tumefaciens, Rhodoferax ferrireducens, Burkholderia multivorans, Xanthobacter autotrophicus, *Roseovarius* sp217, Nitrobacter hamburgensis, Chlorobium phaerobacteroides, Chloroflexus aurentiacus, Thermus thermophilus HB8, Aeropyrum pernix, Sulfolobus tokodai, Environmental sample 1, and Environmental sample 2.

Although arsenic methylation occurs widely in the environment**,** only a single bacterial methyltransferase (ArsM), has been characterized thus far [35]. Neither a homologous *arsM* gene nor arsenic methylation activity was detected in H. arsenicoxydans (unpublished data). In contrast, As[III] oxidation has been demonstrated in this organism [36], resulting from the expression of the *aoxAB* operon (Table 2). The AoxA-Rieske protein-encoding gene is located upstream from the AoxB catalytic subunit gene [36]. Examination of available sequencing data, including those from the Sargasso Sea metagenome [37], suggests a similar organization of putative As[III] oxidase genes in various microorganisms, e.g., Thermus thermophilus, *Chloroflexus aurantiacus,* and Aeropyrum pernix (Figure 3), but not in the facultative autotrophic arsenite-oxidizing bacterium Alkalilimnicola ehrlichei MLHE-1, in which no *aox* gene has been identified thus far [38]. However, comparison of the neighboring CDSs revealed a limited synteny of other genes belonging to the *aoxAB* cluster in most organisms, even though an “arsenic genes island” as defined in Alcaligenes faecalis [10] (i.e., *aoxAB* genes close to arsenate resistance genes *[ars]*), was found in H. arsenicoxydans and in other organisms such as Nitrobacter hamburgensis and Chlorobium phaeobacteroides. Remarkably, the second phosphate-specific transport locus identified in *H. arsenicoxydans,* which shows similarity with phosphate-specific transport systems in Serratia marcescens [39] and in Pseudomonas putida [40], is located in the vicinity of the *aoxRSAB* locus (Figure 3). This supports a link between arsenate, a structural analogue of phosphate, and phosphate transport. Finally, homologs of *aoxRS,* a two-component signal-transduction system identified in Agrobacterium tumefaciens [41], were found upstream from *aoxAB* in H. arsenicoxydans and most of the proteobacteria (Figure 3)*.* Their inactivation by transposon mutagenesis led to a complete loss of arsenite oxidase activity (Figure S3). These genes were, however, not detected in the genome of other As[III]-oxidizing prokaryotes, which suggests important differences in *aoxAB* regulation among microorganisms.

**Figure 4:**
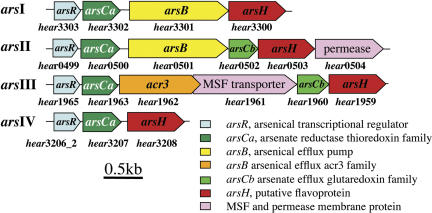
Organization of Arsenate-Resistance Operons *(ars)* in *H. arsenicoxydans* The three operons identified by complementation of an *E. coli ars*-deficient strain code for an ArsR regulator, one or two ArsC arsenate reductases, an As[III] extrusion pump, and an ArsH putative flavoprotein. The fourth operon present in the genome lacks the As[III] pump-encoding gene.

Compared to most microorganisms, including R. metallidurans and the arsenic metabolizer *Alkalilimnicola ehrlichei,* which contain a single *ars* operon, H. arsenicoxydans is remarkable in that its genome harbors four different *ars* loci (Figure 1). Indeed, three clusters of genes involved in resistance to arsenic were identified from a DNA genomic library in complementation experiments with an E. coli strain in which the *ars* operon was deleted. They code for an ArsR regulator, an As[III] extrusion pump, an ArsH putative flavoprotein with no known function [42,43], and one or two arsenate reductases (ArsC), and confer a high level of resistance to arsenic (Figure 4). Moreover, in silico analysis of the genomic data revealed the presence in H. arsenicoxydans of a fourth operon that lacks the As[III] pump-encoding gene and cannot therefore confer resistance to arsenate. Its physiological *raison d'être* remains enigmatic. Quantitative analysis of the transporter-encoding gene mRNA demonstrated that the resistance operons are either constitutively expressed or induced in the presence of As[III] in H. arsenicoxydans (Figure S4; Table 2). These observations were further supported by differential proteomic analyses of the ArsH, ArsC, and ArsR proteins preferentially accumulated on bidimensional gels in the presence of arsenic (Figure S4;Table 2).

**Figure 5:**
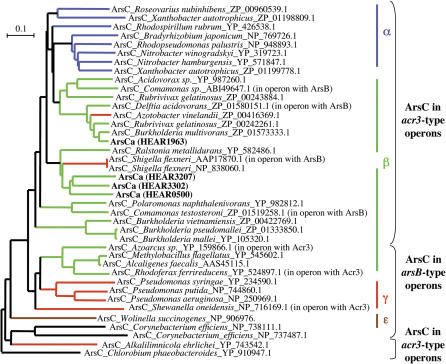
Phylogenetic Tree of ArsCa Arsenate Reductases in Various Arsenic-Resistant Microorganisms The proteins of loci 1 (HEAR3302), 2 (HEAR0500), and 4 (HEAR3207) in cluster with reductases present in *acr3*-type transporter operons. In *H. arsenicoxydans,* two of them are, however, associated with an ArsB-type transporter*.* Protein sequences involved in arsenate reduction were retrieved from the National Center for Biotechnology Information GenBank database (http://www.ncbi.nlm.nih.gov/entrez) and phylogenetic trees were reconstructed from multiple sequence alignments using the neighbor-joining algorithm implemented in ClustalX. The following sequences were used as references: *Roseovarius nubinhibens, Xanthobacter autotrophicus, Rhodospirillum rubrum, Bradyrhizobium japonicum, Rhodopseudomonas palustris, Nitrobacter winogradskyi, Nitrobacter hamburgensis, Xanthobacter autotrophicus, Acidovorax sp., Comamonas sp., Rubrivivax gelatinosus, Delftia acidovorans, Azotobacter vinelandii, Rubrivivax gelatinosus, Burkholderia multivorans, Ralstonia metallidurans, Shigella flexneri, Shigella flexneri, Polaromonas naphthalenivorans, Comamonas testosteroni, Burkholderia vietnamiensis, Burkholderia pseudomallei, Burkholderia mallei, Azoarcus sp., Methylobacillus flagellatus, Alcaligenes faecalis, Rhodoferax ferrireducens, Pseudomonas syringae, Pseudomonas putida, Pseudomonas aeruginosa, Shewanella oneidensis, Wolinella succinogenes, Corynebacterium efficiens, Corynebacterium efficiens, Alkalilimnicola ehrlichei,* and Chlorobium phaeobacteroides.

Four arsenate reductases (ArsCa) in these operons belong to the group typified by the Staphylococcus aureus enzyme [44]. The *arsCa* genes show a high sequence similarity and phylogenetic trees indicate that *arsCa* of loci 1, 2, and 4 arose from a recent gene duplication within the *Herminiimonas* lineage (Figure 5). The same is true for the ArsR regulator and ArsH-encoding genes (unpublished data). Loci 2 and 3 operons additionally harbor a second reductase gene, *arsCb* (Figure 4), which is homologous to that of the E. coli R773 plasmid [45]. An extensive analysis of available bacterial genome data shows that the simultaneous presence of *arsCa* and *arsCb* genes in arsenic resistance operons is common among α, β-, and γ-proteobacteria. The frequent occurence of both *arsC*s in one operon argues against a mere redundancy of functions but is rather in favour of specific roles for each enzyme. The S. aureus ArsC-type enzyme has been shown to use thioredoxin as a reductant [46] whereas the E. coli ArsC-type protein works with glutaredoxin [47]. It therefore seems likely that the association of ArsCa and ArsCb enzymes enables various metabolic pathways to contribute reducing equivalents to the arsenic detoxification reaction, further enhancing the efficiency of this process.

Finally, the membrane transporter proteins differ strongly between the first two loci and locus 3 (Figure 4). The latter contains an Acr3-type transporter [48], which is typical for α-, β-, and several γ-proteobacteria (Figure 5). The other loci both associate an ArsB-type transporter with ArsC reductases that cluster with homologous enzymes usually associated with the Acr3-type transporter. Moreover, ArsB-type efflux pumps seem to be the main rule in Firmicutes, ɛ-, and γ- proteobacteria (Figure 5). H. arsenicoxydans thus stands out among β-proteobacteria by its possession of two ArsB-type transporters in its *ars* operons.

### Regulation and Colonization Functions

Natural isolates have to constantly monitor the physicochemical parameters of their environment, which explains why numerous regulator-encoding genes have usually been identified in their genomes. Except for histone-like nucleoid structuring protein (H-NS), a RNA/DNA-associated protein widespread in proteobacteria [49], the H. arsenicoxydans genome contains a complete set of genes coding for nucleoid-associated proteins such as HU, IHF, FIS, and Hfq. No gene coding for catabolite activator protein was identified, consistent with the lack of carbohydrate metabolism in H. arsenicoxydans. In contrast, the genome codes for the O_2_ responsive protein Fnr, enabling the modulation of the various respiratory pathways [50]. Moreover, 42 genes coding for histidine kinases and response regulators were identified, which correspond to more than 1% of the whole genome. They include those involved in the regulation of the resistance to or the detoxification of toxic metals and metalloids such as arsenic (Figure S3) and presumably copper (Figure S1). The genome also contains genes coding for a QseBC two-component regulatory system known to control flagellum synthesis and motility by quorum sensing. No gene leading to autoinducer synthesis was identified, but this does not rule out the existence of an unidentified quorum-sensing system.

Flagellar genes are mainly clustered at two loci in the chromosome and were shown to encode a polar flagellum (Figure S5 and Table 2). The rotation of this appendix is driven by sodium motive force, as demonstrated by the loss of motility of H. arsenicoxydans in the presence of 0.3 mM amiloride, an inhibitor of Na^+^/H^+^ antiporters (Figure S6). Surprisingly, the synteny in the first locus is highly reminiscent of that in E. coli, whose motility is known to depend on peritrichous flagella [51]. This region contains the *flhDC* master operon in H. arsenicoxydans, suggesting that the flagellum-encoding genes are organized in a mixed peritrichous/polar cascade in this organism. Such a novel hierarchical cascade most probably results from gene acquisition from multiple sources followed by DNA rearrangements.

**Figure 6:**
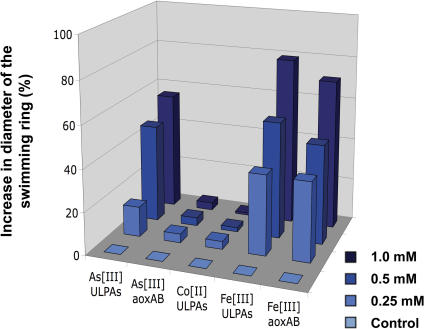
Effect of Metal and Metalloid Concentration on Swimming Properties in H. arsenicoxydans Motility assays were performed in the presence of an increasing concentration of As[III], Co[II], or Fe[III]. The level of motility of ULPAs wild-type strain and of its *aoxAB* knockout derivative was evaluated as the diameter of the swimming ring expressed in millimeters. The results are the mean value of three independent experiments.

**Figure 7:**
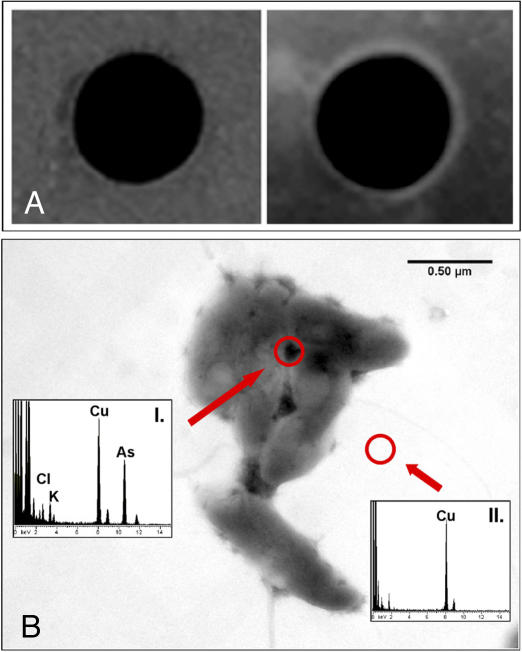
Chemotaxis and EPS Synthesis in H. arsenicoxydans in Response to Arsenic (A) Chemoattraction to As[III]. Left: low melting agar with chemotaxis buffer, right: low melting agar with chemotaxis buffer supplemented with 2 mM As[III]. Bright ring of cells around agarose plugs is indicative of chemotaxis. (B) Transmission electron microscopy (TEM) picture of H. arsenicoxydans grown in As-enriched medium. Circles represent the X-ray spot of analysis, while I and II are the energy dispersive X-Ray spectroscopy corresponding values. Cl and K peaks show organic constituents and Cu labels represent peaks due to supporting grid. Arsenic content is 16.5 % weight as As_2_O_3_ in I. and 0.0% weight in II; both including microgrid C-coating quantification.

Although the flagellum morphology was not affected by the presence of arsenic (Figure S5), at least with respect to length and width, an increased concentration of this toxic element resulted in a concomitant increase in bacterial motility on semisolid agar plates (Figure 6) and in flagellar gene expression (Table 2). Aside from iron, an element that is essential to life, no such effect occurred with other toxic elements tested, such as Co[II] (Figure 6), Cu[II], Sb[III], or As[V] (unpublished data). The hypothesis that arsenic contributes to the metabolism of H. arsenicoxydans was further supported by the positive chemotactic response shown by the strain towards As[III] (Figure 7A). This observation suggests that the bacterium is able to sense and respond to the presence of As[III] in the medium. The genome of H. arsenicoxydans contains 12 methyl-accepting chemotaxis proteins–encoding genes. As most of these genes have no predicted function, it is tempting to speculate that at least one of them plays a role in this mechanism.

To determine how arsenic contributes to motility, GFP strains were constructed and their swimming behaviour was studied using video microscopy methods. While the average swimming speed of H. arsenicoxydans was 30 μm/s, a 2-fold increase was observed in the presence of 2 mM As[III]. Disruption of *aoxA* or *aoxB* gene by a transposon insertion located at the 84th or the 335th codon, respectively, abolished the improvement in the swimming performances in the presence of As[III] but not in the presence of Fe[III] (Figure 6), which suggests that the strain may gain additional energy from the arsenic-oxidation process. The presence of *aoxD,* a cytochrome *c*552–encoding gene, in the vicinity of the *aoxAB* operon (Figure 3) further supports this hypothesis.

Finally, in contrast to related β-proteobacteria such as R. metallidurans, the genome of H. arsenicoxydans contains a type IV pilin gene cluster. This mannose-sensitive haemagglutinin-encoding gene may be of importance in the interaction between H. arsenicoxydans and the microflora present in its environment. Moreover, electron microscopy examination revealed the induction of a thick capsule when H. arsenicoxydans was cultivated in As[III]-containing medium (Figure S5). An operon of 18 genes present in the genome, induced in response to arsenic (Table 2) and possibly involved in the synthesis of exopolysaccharides (EPS), may play a role in this process. In addition, nanoparticles were shown to accumulate in the capsule of H. arsenicoxydans as compared to H. fonticola, a phylogenetically related strain that does not oxidize arsenic (Figure S7). Physicochemical analysis by transmission electron microscopy/energy dispersive X-Ray spectroscopy demonstrated the existence of a high arsenic content, suggesting for the first time a role for EPS in the scavenging of this toxic element (Figure 7B).

## Discussion

Within the Oxalobacteraceae family, H. arsenicoxydans and the closely related strains H. aquatilis and *H. fonticola* represent a new genus comprising bacteria isolated from diverse aquatic environments. Microorganisms of this novel taxonomic group may therefore be widespread in such natural or anthropized ecosystems. The genome sequence and the physiology of H. arsenicoxydans further support the ability of this strain to grow in a wide range of environmental conditions, in particular with respect to oxygen concentrations. Moreover, genomic and experimental data demonstrated that this organism is capable of accommodating the presence of high concentrations of various toxic metals. More importantly, H. arsenicoxydans has evolved multiple processes not only to resist arsenic toxicity, such as DNA repair, oxidative stress resistance, and As[III] extrusion, but also to detoxify it for its own profit, such as As[III] oxidation and its probable involvement in energy metabolism. Some of these unusual genetic determinants, acquired most probably by horizontal gene transfer, are organized as genomic islands.

Remarkably, the versatile regulatory system of H. arsenicoxydans enables it to sense dynamic changes in arsenic concentration and to initiate motility and EPS synthesis for attachment to this metalloid. Such adaptive mechanisms may play a key role in the environment, allowing microorganims to efficiently flourish and colonize arsenic-rich ecosystems. Moreover, recent results suggest that microbial biofilms are involved in the adsorption and immobilization of metals such as Pb[II] [52] and Cr[III] [53]. These properties have been used in biorehabilitation of aqueous solutions contaminated with heavy metals [54]. The ability of H. arsenicoxydans to scavenge arsenic in an EPS matrix may be of prime importance in the context of bioremediation of contaminated environments, leading to the sequestration of this toxic metalloid.

To our knowledge, the genome of H. arsenicoxydans is the first to be fully characterized for an arsenic-metabolizing microorganism. The results presented here provide evidence that the ability of microbes to colonize arsenic-rich environments extends beyond the biotransformation of this toxic element. Although biochemical processes play an important role in arsenic release into the environment [7,8], the physiology of the microbes inhabiting extreme ecological niches may not be restricted solely to oxidoreduction reactions. In this respect, the positive chemotaxic response to the presence of arsenic and the scavenging of this element, associated with the transformation of As[III] into its less toxic form As[V], may have been key mechanisms in the colonization of the ancient environment on earth, allowing for the development of other microorganisms. In the near future, sequencing data for other arsenic-metabolizing organisms, combined with molecular biology, genetics, biochemistry, and biophysics approaches, will lead us to identify new arsenic-dependent processes. H. arsenicoxydans may therefore constitute a reference bacterium for further research towards a comprehensive analysis of the molecular mechanisms governing biological arsenic responses.

## Materials and Methods

### Genome sequencing, assembly, and annotation.

The complete genome sequence of H. arsenicoxydans was determined using the whole-genome shotgun method. Three genomic libraries were constructed, i.e., two plasmid libraries (obtained after mechanical shearing of DNA and cloning of generated 3–4 kb and 8–10 kb fragments into plasmids pcDNA2.1 (Invitrogen, http://www.invitrogen.com) and pCNS (a pSU18-derivative), respectively, and one BAC library to order contigs (obtained by partial digestion with *Sau3*A of the genomic DNA and the introduction of ∼20-kb fragments into pBeloBac11 (New England Biolabs, http://www.neb.com). From these libraries, 26,112, 7,680 and 3,840 clones, respectively, were end-sequenced using dye-terminator chemistry on ABI3730 sequencers (Applied Biosytems, http://www.appliedbiosystems.com). The Phred/Phrap/Consed software package (http://www.phrap.com) was used for sequence assembly and quality assessment [55–57]. About 732 additional reactions were necessary to complete the genomic sequence.

Using the AMIGene software (Annotation of MIcrobial Genes) [58], a total of 3,355 CDSs were predicted (and assigned a unique identifier prefixed with “HEAR”) and submitted to automatic functional annotation: BLAST searches against the UniProt database (http://www.uniprot.org) were performed to determine significant homology. Based on the biological representation of the translation process, we applied Bayesian statistics to create a score function for predicting translation start sites. We integrated together the ribosome binding site sequence, the distance between the translation start site and the ribosome binding site sequence, the base composition of the start codon, the nucleotide composition following start codons, and the expected distribution of proteins length. To further increase the prediction accuracy, we took into account the predicted operon structures. These elements were combined to create a score function and the highest score was selected for the translation start site predictions (Y. Makita, unpublished data). Protein motifs and domains were documented using the InterPro database (http://www.ebi.ac.uk/interpro). In parallel, genes coding for enzymes were classified using the PRIAM software [59]. TMHMM v. 2.0 was used to identify transmembrane domains [60], and SignalP 3.0 was used to predict signal peptide regions [61]. Finally, tRNAs were identified using tRNAscan-SE [62]. Sequence data for comparative analyses were obtained from the NCBI database (RefSeq section, http://www.ncbi.nlm.nih.gov/RefSeq). Putative orthologs and synteny groups (i.e., conservation of the chromosomal colocalisation between pairs of orthologous genes from different genomes) were computed between H. arsenicoxydans and all the other complete genomes as previously described [63]. Manual validation of the automatic annotation was performed using the MaGe (Magnifying Genomes, http://www.genoscope.cns.fr) interface, which allows graphic visualization of the H. arsenicoxydans annotations enhanced by a synchronized representation of synteny groups in other genomes. The H. arsenicoxydans nucleotide sequence and annotation data have been deposited in the EMBL database (http://www.ebi.ac.uk/embl; see accession numbers list below).

All these data (i.e., syntactic and functional annotations, and results of comparative analysis) were stored in a relational database, called ArsenoScope [63]. This database is publicly available via the MaGe interface at https://www.genoscope.cns.fr/agc/mage.

### Genetic and molecular biology.

Mutations in genes induced by arsenic or mutant strains expressing GFP were obtained by random insertion of a mini-Tn*5* and of a mini-Tn*5* harboring a modified GFP-encoding gene [64], respectively. DNA manipulation and sequence analysis were performed as previously described [36]. These analyses showed that the KDM-7 *gfp* mutant strain used in the present study carries a mini-Tn*5* insertion in *hear1692,* a CDS coding for a conserved hypothetical protein. RNA preparation, probe construction, and quantitative analysis of transcripts were performed as previously described [65].

The H. arsenicoxydans genomic library was constructed in plasmid pcDNA 2.1 (Invitrogen) as previously described [66]. About 60,000 clones were selected on LB plates supplemented with 100 μg/ml ampicillin and pooled. Large-scale plasmid DNA isolation was carried out using the JETstar kit (GenoMed, http://www.genomed.com). This library was used to transform the E. coli arsenic-susceptible strain AW3110. Clones of interest were selected on LB medium supplemented with 5mM AS[III].

### Microbiological methods.

Strains were cultivated in a chemically defined medium (CDM) or in tryptone medium at 25 °C supplemented with 1.5% agar when required [12]. Minimal inhibitory concentrations and arsenite oxidase activity were determined on agar plates as previously described [36]. CDM supplemented with 1% tryptone and 0.3% agar was used to test bacterial motility as previously described [67]. Bacterial cell tracking was performed as follows: ULPAs1 strain was grown in CDM for 72 h and 0.5 ml of the cell culture was then transferred into 4.5 ml of fresh CDM medium containing 1% tryptone. Half of the cell culture was further incubated for 24h in the presence of 50ppm As[III], and the other half was incubated without As[III]. 30 min prior to the cell tracking procedure, 100ppm As[III] was added to the samples incubated with arsenic.

Bacterial survival after UV treatment was determined using a spot-plating technique [68]. Serially diluted cultures (10- to 10^9^-fold) were spotted (5 μl) in triplicate onto CDM agar plates. Plates were then incubated for 72 h at 25 °C in the dark. Dilution with spots containing five to 30 colonies were counted, and the resulting average was expressed as the ratio of viable cells in treated cultures to those in untreated cultures. The wild-type strain H. arsenicoxydans and mutants strains *polA*::Tn*5, recQ*::Tn*5,* and *radA*::Tn*5* were grown in CDM or CDM supplemented with 300ppm As[III]. Plates were irradiated for different lengths of time with UV light (254 nm) from a germicidal lamp at a rate of 5 J m^−2^ s^−1^ and then incubated in the dark as described above.

Chemotaxis assays were performed using a modified agarose plug method [69]. The sides of the chemotaxis chamber each consisted of two plastic strips 15 mm apart placed on a glass slide. A drop of molten low melting point agarose containing the substrate to be tested was placed on a coverslip that was reversed onto the plastic strips to form a chamber. Cells intended for chemotaxis assays were grown in the presence of arsenite As[III], collected, resuspended in chemotaxis buffer, and supplied with 0.5 mM acetate as an energy source before being flooded into the chamber.

### Two-dimensional electrophoresis, mass spectrometry, and protein identification.

Isoelectric focusing was conducted using the horizontal Multiphor II system (Pharmacia) from 60 μg of protein loaded onto an 18 cm pH 4–7 immobilized pH gradient strip. To account for unspecific variations, eight gels—obtained by using two independent protein preparations extracted from four independent cultures—were run for each condition (in the presence or in the absence of arsenite). In-gel protein digestion of the spots was performed with an automated protein digestion system, MassPREP Station (Waters, http://www.waters.com), and the resulting peptide extracts were then directly analysed by nanoLC-MS/MS on an Agilent 1100Series capillary LC system (Agilent Technologies, http://www.home.agilent.com) coupled to a HCT Plus ion trap (Bruker Daltonics, http://www.bdal.com/) as previously described [65].

Protein identifications were performed directly in the uninterpreted genome database. The complete genome sequence of H. arsenicoxydans was fragmented in regular length segments of 7,500 bases with 2,000 overlapping bases, translated in the six possible reading frames, and imported into a local Mascot (Matrix Science, http://www.matrixscience.com) proteomic search engine. The matching peptides thus allowed the identification of the coding region directly on the genome sequence. These matching peptides were then exported to the MS-BLAST program [70] to identify the function of the proteins by homology with proteins of organisms that are present in the databases. Aside from the identification of proteins differentially regulated in the presence of arsenic, a large proteome map was generated. These proteomic results were compared to the computational genome annotation results, which allowed the correction of several translation start codons, therefore constituting an additional validation tool for computational predictions.

Steps of the interpretation were automated and compiled thanks to homemade software and the interactive proteomic map of H. arsenicoxydans is publicly available via the InPact interface at http://inpact.u-strasbg.fr.

### Electron and fluorescence microscopy.

Bacterial cells were grown in CDM medium and stained with 0.1% (v/v) osmium tetroxide prepared in water as previously described [12].

*H. arsenicoxydans gfp* cells were tracked as follows: 600 μl of each cell suspension was poured into a well and the cells were excited with UV light for 200 ms. The image acquisition was performed with a Nikon 2000 Eclipse epifluorescence microscope (http://www.nikonusa.com) equipped with a CCD camera and gray scale 16-bit images were recorded. The frame of the images was 100 μm in size. The digitized fluorescent signals emitted by GFP-positive cells were taken every 243 ms during 25-s time periods. These sequences of images were further analyzed in the form of stacks using the image analysis software package ImageJ (http://rsb.info.nih.gov/ij). The swimming speed was measured in the absence or the presence of sublethal arsenic concentration as follows: all pictures in each dynamic image sequence (stack) were normalized by subtracting the background and the resulting image was run through a sharpening edges filter. The signal was then enhanced and a crosshair (mark and count) tool was used to manually mark each displacement of a given cell through the stack. A manual threshold was applied to remove any remaining background noise and all the stacks were transformed into 8-bit binarized images. The distance traveled by a cell between two positions (from one image to another) was calculated using a 2-D coordinate system. The (x, y) coordinates of the bacterial cells were evaluated and the distance traveled was calculated according to the following equation:

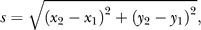
where *y*_1_ and *y*_2_ are the positions of the marked pixels (cell) on the y axis and *x*_1_ and *x*_2_, those on the x axis in two adjacent images. The time taken by the bacteria to perform this movement was calculated as the number of images containing the bacterium (traced frames) divided by the frame capture interval of four pictures per second, i.e., t = 4 * N_traces_. All the displacements of each tracked cell were then combined and the average speed of each cell in μm/s was calculated using the velocity formula 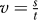 . The final cost function giving the average velocity of a single cell was:

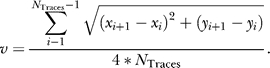



Three separate cell cultures, each in triplicate samples, were assayed. For each sample, three stacks of images were analyzed.

Physicochemical analysis of the nanoparticles contained in the capsule of H. arsenicoxydans were performed using transmission electron microscope/energy dispersive X-ray spectroscopy). In order to provide such observations, bacteria were grown in enriched arsenic medium for 48 hours. A 6 μl drop of 20-fold diluted suspension was put onto a C-coated copper microgrid and air-dried. The transmission electron microscope used is a Philips STEM 420 (http://www.research.philips.com) operated at 120 kV. Qualitative and quantitative analyses were carried out using an INCA (Oxford Instruments, http://www.oxford-instruments.com) X-ray microanalyser. Analyses demonstrated that nanoparticles contain a high amount of arsenic.

## Supporting Information

Figure S1 Comparative Chromosomal Arrangement of Metal Resistance Cluster Genes in H. arsenicoxydans and R. metallidurans
The synteny region between two species was defined to include both conservation of the gene content and order. Genes in the R. metallidurans genome were predicted from the DOE Joint Genome Institute data. In the absence of complete functional studies, in particular the detection of transcription terminators, we assume that each cluster is represented by the longest DNA segment delimited by conserved ORFs in at least one of the clusters. The genes present in each cluster are indicated by arrows (not drawn to scale) indicating the direction of transcription. The genes of conserved synteny are identified by color coding and labeled with the annotation gene names.
(A) Cadmium-zinc-cobalt–resistance operons.
(B) Copper-resistance operons.
(C) Chromate-resistance operons.
(2.5 MB JPG)

Figure S2 Arsenic Stress and DNA Repair (A) Effect of arsenic on gene expression. The expression of several genes known to play a role in DNA recombination and repair and/or stress adaptation (Table 2) was evaluated by comparing their mRNA content by slot-blotting hybridisation experiments after exposure to 300 ppm As[III] for 15 min. The ribosomal *rrn* mRNA was used as a control.
(B) Survival of DNA repair and recombination Tn*5* mutants after UV irradiation in the presence or absence of arsenic. To evaluate the effect of UV exposure on the survival of strains mutated in *polA, radA,* or *recQ*, plates were irradiated for different times with UV light (254 nm) from a germicidal lamp at a rate of 5 J m^−2^ s^−1^ and then incubated in the dark. In addition, to determine the possible concomitant effect of arsenic, cells subjected to UV irradiation were incubated in the presence of 300 ppm As [III]. In each case, survival was evaluated by determination of colony forming units (CFU) on CDM medium. The results are representative of 3 independent experiments.
(190 KB JPG)

Figure S3 Arsenite Oxidase Activity in H. arsenicoxydans Wild-Type and Mutant Strains Strains tested for oxidation of arsenite to arsenate were grown for 24 h at 25 °C on CDM agar plates supplemented with arsenite (100 mg/l). Agar plates were then flooded with a solution of 0.1 M AgNO_3_. A brownish precipitate revealed the presence of arsenate in the medium surrounding the *H. arsenicoxydans* wild-type strain as compared to the *aoxA, aoxB,* and *aoxS* mutants carrying a Tn*5* insertion located at the 84^th^, 335^th^ codon, and 268^th^ codon, respectively.
(1.1 MB JPG)

Figure S4 Expression of *ars* Operons and Synthesis of the Corresponding Proteins in Response to Arsenic (A) Effect of arsenic on *ars* operon expression. The level of expression was evaluated by determining the mRNA content of the transporter-encoding gene, i.e., the *arsB* pump in operons I and II and *acr3* transporter in operon 3, by slot-blotting hybridisation experiments. The ribosomal *rrn* gene was used as a control. Similar results were obtained by RT-PCR analysis of the *arsCa* gene mRNA (unpublished data), also suggesting that expression of the fourth operon was also induced by arsenic.
(B) Effect of arsenic on *ars* protein synthesis. ULPAs wild-type strain was cultured in the presence of 300 ppm As[III] and proteins preferentially accumulated on 2D gels as compared to those of the same strain cultured in arsenic-free medium were identified by nanoLC-MS/MS analysis. A subset of *ars* operon proteins are circled in red. A complete protein map of H. arsenicoxydans is publicly available via the InPact interface at http://www.inpact.u-strasbg.fr.
(1.4 MB JPG)

Figure S5 Polar Flagellum and EPS Synthesis in H. arsenicoxydans in the Presence of Arsenic Bacterial cells were grown with moderate shaking in CDM medium supplemented (right) or not (left) with 300 ppm As[III] and stained with 0.1% (v/v) osmium tetroxyde prepared in water. Cells (20 μl) were deposited onto 300-mesh copper grids coated with Formvar (Electron Microscopy Sciences, http://www.emsdiasum.com/ems) and examined at 75 keV under a Hitachi H600 transmission electron microscope (http://www.hitachi.com). Images acquisition was performed with a CCD Advantage HR Hamamatsu camera (http://www.hamamatsu.com)and the AMT 542 software (Advanced Microscopy Techniques, http://www.amtimaging.com).
(1.1 MB JPG)

Figure S6 Swimming Properties of H. arsenicoxydans Wild-Type and Mutant Strains The motility assay was performed on semi-solid medium plates. The results are representative of three independent experiments. As compared to the wild-type strain (left), no motility was observed in the presence of 0.3 mM amiloride (middle) or with the mutant strain (right) carrying a Tn*5* tranposon insertion in the 88th codon of the *fliL* gene [65]
(783 KB JPG)

Figure S7 Arsenic Scavenging by EPS in the Presence of Arsenic Transmission electron microscopy (TEM) picture of H. arsenicoxydans (left) and of H. fonticola (right), an arsenic non-oxidizing related strain, grown in the presence of 25 ppM As[III]. Bacterial cells were cultivated in CDM medium and stained with 0.1% (v/v) osmium tetroxide prepared in water. Red arrows indicate the presence of As particles in the EPS matrix of *H. arsenicoxydans.*
(613 KB JPG)

Table S1 Insertion Sequences Identified in the Herminiimonas arsenicoxydans Genome Only ISs with at least one complete copy in the genome are named (http://www-is.biotoul.fr).
(48 KB DOC)

Table S2 Description of the H. arsenicoxydans GC-Rich Island Locus tag, gene name, and description for H. arsenicoxydans are colored according to Figure 2 (red for arsIII cluster, blue for the part of the *clc* element, and green for the more specific metabolic capabilities found in the third part of this island). For each of the proteobacteria compared, the CDS locus tag and the percent identity of the match between the two proteins are given using the color scheme found in the synteny map (see Figure 2). Only orthologs in synteny with H. arsenicoxydans genes are listed; comments in black provide a description of the functions encoded by genes of the compared genomes that are not similar to H. arsenicoxydans genes in this GC-rich island.
(55 KB XLS)

Table S3 Carbon and Energy Metabolism in Herminiimonas arsenicoxydans
(62 KB DOC)

Table S4 Minimal Inhibitory Concentration of Toxic Ions in Herminiimonas arsenicoxydans ULPAs1, H. fonticola S94, and Ralstonia metallidurans CH34. H. fonticola S94, which has been isolated from a non-contaminated environment, was used as a control.
(41 KB DOC)

### Accession Numbers

The EMBL (http://www.ebi.ac.uk/embl) accession number for the genome of H. arsenicoxydans is CU207211.

Sequence information corresponding to genes or proteins identified in other genomes and presented in Figures 2, 3, and 5, respectively, was obtained from the National Center for Biotechnology Information GenBank database (http://www.ncbi.nlm.nih.gov/entrez) under the following accession numbers:

Figure 2 accession numbers: Azoarcus sp. EbN1 (NC_006513), Burkholderia xenovorans LB400 (NC_007951), Pseudomonas fluorescens Pf-5 (NC_004129), Ralstonia metallidurans CH34 (NC_007973), and Xanthomonas campestris 85–10 (NC_007508).

Figure 3 accession numbers: Aeropyrum pernix (NC_000854), Agrobacterium tumefaciens (DQ151549), Alcaligenes faecalis (AY297781), Burkholderia multivorans (NZ_AAVB01000013), Chlorobium phaero-bacteroides (NZ_AAIC01000001), Chloroflexus aurantiacus (NZ_AAAH02000003), Environmental sample 1(AACY01058630), Environmental sample 2 (AACY01082423), Nitrobacter hamburgensis (NC_007960.1), Rhodoferax ferrireducens (NC_007908), *Roseovarius* sp217 (NZ_AAMV01000002.1), Sulfolobus tokodai (BA000023.2), Thermus thermophilus HB8 (NC_006462), and Xanthobacter autotrophicus (NZ_AAPC01000006).

Figure 5 accession numbers: Acidovorax sp. (YP_987260.1), Alcaligenes faecalis (AAS45115.1), Alkalilimnicola ehrlichei (YP_743542.1), Azoarcus sp. (YP_159866.1), Azotobacter vinelandii (ZP_00416369.1), Bradyrhizobium japonicum (NP_769726.1), Burkholderia mallei (YP_105320.1), Burkholderia multivorans (ZP_01573333.1), Burkholderia pseudomallei (ZP_01333850.1), Burkholderia vietnamiensis (ZP_00422769.1), Chlorobium phaeobacteroides (YP_910947.1), Comamonas sp. (ABI49647.1), Comamonas testosteroni (ZP_01519258.1), Corynebacterium efficiens (NP_737487.1), Corynebacterium efficiens (NP_738111.1), Delftia acidovorans (ZP_01580151.1), Methylobacillus flagellatus (YP_545602.1), Nitrobacter hamburgensis (YP_571847.1), Nitrobacter winogradskyi (YP_319723.1), Polaromonas naphthalenivorans (YP_982812.1), Pseudomonas aeruginosa (NP_250969.1), Pseudomonas putida (NP_744860.1), Pseudomonas syringae (YP_234590.1), Ralstonia metallidurans (YP_582486.1), Rhodoferax ferrireducens (YP_524897.1), Rhodopseudomonas palustris (NP_948893.1), Rhodospirillum rubrum (YP_426538.1),Roseovarius nubinhibens (ZP_00960539.1), Rubrivivax gelatinosus (ZP_00242261.1), Rubrivivax gelatinosus (ZP_00243884.1), Shewanella oneidensis (NP_716169.1), Shigella flexneri (AAP17870.1), Shigella flexneri (NP_838060.1), Wolinella succinogenes (NP_906976.1), Xanthobacter autotrophicus (ZP_01198809.1), and Xanthobacter autotrophicus (ZP_01199778.1)

## Abbreviations

CDM: chemically defined medium
CDS: coding sequence
EPS: exopolysaccharide
IS: insertion sequences
nb: number

## References

[pgen-0030053-b001] Vaughan DJ (2006). Arsenic. Elements.

[pgen-0030053-b002] Jackson CR, Dugas SL (2003). Phylogenetic analysis of bacterial and archaeal *arsC* gene sequences suggests an ancient, common origin for arsenate reductase. BMC Evol Biol.

[pgen-0030053-b003] Lebrun E, Brugna M, Baymann F, Muller D, Lièvremont D (2003). Arsenite oxidase, an ancient bioenergetic enzyme. Mol Biol Evol.

[pgen-0030053-b004] Oremland RS, Kulp TR, Blum JS, Hoeft SE, Baesman S (2005). A microbial arsenic cycle in a salt-saturated, extreme environment. Science.

[pgen-0030053-b005] Ouzounis CA, Kunin V, Darzentas N, Goldovsky L (2006). A minimal estimate for the gene content of the last universal common ancestor—exobiology from a terrestrial perspective. Res. Microbiol.

[pgen-0030053-b006] Smith AH, Lopipero PA, Bates MN, Steinmaus CM (2002). Public health. Arsenic epidemiology and drinking water standards. Science.

[pgen-0030053-b007] Oremland RS, Stolz JF (2003). The ecology of arsenic. Science.

[pgen-0030053-b008] Islam FS, Gault AG, Boothman C, Polya DA, Chamok JM (2004). Role of metal-reducing bacteria in arsenic release from Bengal delta sediments. Nature.

[pgen-0030053-b009] Anderson GL, Williams J, Hille R (1992). The purification and characterization of arsenite oxydase from Alcaligenes faecalis, a molybdenum-containing hydroxylase. J Biol Chem.

[pgen-0030053-b010] Silver S, Phung LT (2005). Genes and enzymes involved in bacterial oxidation and reduction of inorganic arsenic. Appl Environ Microbiol.

[pgen-0030053-b011] Simeonova DD, Micheva K, Muller DAE, Lagarde F, Lett MC (2005). Arsenite oxidation in batch reactors with alginate-immobilized ULPAs1 strain. Biotech Bioeng.

[pgen-0030053-b012] Muller D, Simeonova DD, Riegel P, Mangenot S, Koechler S (2006). Herminiimonas arsenicoxydans sp. nov., a metalloresistant bacterium. Int J Syst Evol Microbiol.

[pgen-0030053-b013] Handrick R, Reinhardt S, Focarete ML, Scandola M, Adamus G (2001). A new type of thermoalkalophilic hydrolase of Paucimonas lemoignei with high specificity for amorphous polyesters of short chain-length hydroxyalkanoic acids. J Biol Chem.

[pgen-0030053-b014] de Boer W, Leveau JHJ, Kowalchuk GA, Gunnewiek PJAK, Abeln ECA (2004). Collimonas fungivorans gen. nov., sp. nov., a chitinolytic soil bacterium with the ability to grow on living fungal hyphae. Int J Syst Evol Microbiol.

[pgen-0030053-b015] Fernandes C, Rainey FA, Fernanda Nobre M, Pinhal I, Folhas F (2005). Herminiimonas fonticola gen. nov., sp. nov., a Betaproteobacterium isolated from a source of bottled mineral water. Syst Appl Microbiol.

[pgen-0030053-b016] Kämpfer P, Busse HJ, Falsen E (2006). Herminiimonas aquatilis sp. nov., a new species from drinking water. Syst Appl Microbiol.

[pgen-0030053-b017] de la Cruz F, Davies J (2000). Horizontal gene transfer and the origin of species: Lessons from bacteria. Trends Microbiol.

[pgen-0030053-b018] Mergeay M, Monchy S, Vallaeys T, Auquier V, Benotmane A (2003). Ralstonia metallidurans, a bacterium specifically adapted to toxic metals: Towards a catalogue of metal-responsive genes. FEMS Microbiol Rev.

[pgen-0030053-b019] Tuffin M, Hector SB, Deane SM, Rawlings DE (2006). Resistance determinants of a highly arsenic-resistant strain of Leptospirillum ferriphilum isolated from a commercial biooxidation tank. Appl Environ Microbiol.

[pgen-0030053-b020] Dobrindt U, Hochhut B, Hentscheland U, Hacker J (2004). Genomic islands in pathogenic and environmental microorganisms. Nat Rev Microbiol.

[pgen-0030053-b021] Dorn E, Hellwig M, Reineke W, Knackmuss HJ (1974). Isolation and characterisation of a 3-chlorobenzoate degrading pseudomonad. Arch Microbiol.

[pgen-0030053-b022] Gaillard M, Vallaeys T, Vorhölter FJ, Minoia M, Werlen C (2006). The *clc* element of Pseudomonas sp. strain B13, a genomic island with various catabolic properties. J Bacteriol.

[pgen-0030053-b023] Pohlmann A, Fricke WF, Reinecke F, Kusian B, Liesegang H (2006). Genome sequence of the bioplastic-producing “Knallgas” bacterium Ralstonia eutropha H16. Nat Biotechnol.

[pgen-0030053-b024] Cervantes C, Ji G, Ramirez JL, Silver S (1994). Resistance to arsenic compounds in microorganisms. FEMS Microbiol Rev.

[pgen-0030053-b025] van Veen HW (1997). Phosphate transport in prokaryotes: Molecules, mediators and mechanisms. Antonie Van Leeuwenhoek.

[pgen-0030053-b026] Weeger W, Lievremont D, Perret M, Lagarde F, Hubert JC (1999). Oxidation of arsenite to arsenate by a bacterium isolated from an aquatic environment. Biometals.

[pgen-0030053-b027] Pitcher RS, Watmough NJ (2004). The bacterial cytochrome *cbb3* oxidases. BBA-Bioenergetics.

[pgen-0030053-b028] Schneider D, Schmidt CL (2005). Multiple Rieske proteins in prokaryotes: Where and why?. BBA-Bioenergetics.

[pgen-0030053-b029] Smith M, Finel M, Korolik V, Mendz G (2000). Characteristics of the aerobic respiratory chains of the microaerophiles Campylobacter jejuni and Helicobacter pylori. Arch Microbiol.

[pgen-0030053-b030] Hooper AB, Arciero DM, Bergmann D, Hendrich MP, Zannoni D (2004). The oxidation of ammonia as an energy source in bacteria. Respiration in Archaea and Bacteria.

[pgen-0030053-b031] Malasarn D, Saltikov CW, Campbell KM, Santini JM, Hering JG (2004). *arrA* is a reliable marker for As(V) respiration. Science.

[pgen-0030053-b032] Rodriguez-Gabriel MA, Russell P (2005). Distinct signaling pathways respond to arsenite and reactive oxygen species in Schizosaccharomyces pombe. Eukaryot Cell.

[pgen-0030053-b033] Moore LE, Pfeiffer R, Warner M, Clark M, Skibola C (2005). Identification of biomarkers of arsenic exposure and metabolism in urine using SELDI technology. J Biochem Mol Tox.

[pgen-0030053-b034] Smith JL (2004). The physiological role of ferritin-like compounds in bacteria. Critic Rev Microbiol.

[pgen-0030053-b035] Qin J, Rosen BP, Zhang Y, Wang G, Franke S (2006). Arsenic detoxification and evolution of trimethylarsine gas by a microbial arsenite S-adenosylmethionine methyltransferase. Proc Natl Acad Sci U S A.

[pgen-0030053-b036] Muller D, Lièvremont D, Simeonova D. D, Hubert JC, Lett MC (2003). Arsenite oxidase *aox* genes from a metal-resistant β-proteobacterium. J Bacteriol.

[pgen-0030053-b037] Venter JC, Remington K, Heidelberg JF, Halpern AL, Rusch D (2004). Environmental genome shotgun sequencing of the Sargasso Sea. Science.

[pgen-0030053-b038] Oremland RS, Hoeft SE, Santini JM, Bano N, Hollibaugh RA ( 2002). Anaerobic oxidation of arsenite in Mono Lake Water and by a facultative arsenite-oxidizing chemoautotroph, strain MLHE-1. Appl Environ Microbiol.

[pgen-0030053-b039] Lee SJ, Lee YS, Lee YC, Choi YL (2006). J Basic Microbiol.

[pgen-0030053-b040] Wu H, Kosaka H, Kato J, Kuroda A, Ikeda T (1999). Cloning and characterization of Pseudomonas putida genes encoding the phosphate-specific transport system. J Biosc Bioeng.

[pgen-0030053-b041] Kashyap DR, Botero LM, Franck WL, Hassett DJ, McDermott TR (2006). Complex regulation of arsenite oxidation in Agrobacterium tumefaciens. J Bacteriol.

[pgen-0030053-b042] Butcher BG, Deane SM, Rawlings DE (2000). The chromosomal arsenic resistance genes of Thiobacillus ferrooxidans have an unusual arrangement and confer increased arsenic and antimony resistance to Escherichia coli. Appl Environ Microbiol.

[pgen-0030053-b043] Lopez-Maury L, Florencio FJ, Reyes JC (2003). Arsenic sensing and resistance system in the cyanobacterium Synechocystis sp. strain PCC 6803. J Bacteriol.

[pgen-0030053-b044] Zegers I, Martins JC, Willem R, Wyns L, Messens J (2001). Arsenate reductase from S. aureus plasmid pI258 is a phosphatase drafted for redox duty. Nat Struct Biol.

[pgen-0030053-b045] Martin P, DeMel S, Shi J, Gladysheva T, Gatti DL (2001). Insights into the structure, solvation, and mechanism of ArsC arsenate reductase, a novel arsenic detoxification enzyme. Structure.

[pgen-0030053-b046] Ji G, Garber EA, Armes LG, Chen CM, Fuchs JA (1994). Arsenate reductase of Staphylococcus aureus plasmid pI258. Biochemistry.

[pgen-0030053-b047] Liu J, Rosen BP (1997). Ligand interactions of the ArsC arsenate reductase. J Biol Chem.

[pgen-0030053-b048] Rosen BP (1999). Families of arsenic transporters. Trends Microbiol.

[pgen-0030053-b049] Tendeng C, Bertin PN (2003). H-NS in Gram-negative bacteria: A family of multifaceted proteins. Trends Microbiol.

[pgen-0030053-b050] Korner H, Sofia HJ, Zumft WG (2003). Phylogeny of the bacterial superfamily of Crp-Fnr transcription regulators: exploiting the metabolic spectrum by controlling alternative gene programs. FEMS Microbiol Rev.

[pgen-0030053-b051] Soutourina OA, Bertin PN (2003). Regulation cascade of flagellar expression in Gram-negative bacteria. FEMS Microbiol Rev.

[pgen-0030053-b052] Templeton A, Spormann A, Brown G (2003). Speciation of Pb(II) sorbed by Burkholderia cepacia/goethite composite. Environ Sci Technol.

[pgen-0030053-b053] Kang S, Bremer P, Kim K, McQuillan A (2006). Monitoring metal ion binding in single–layer Pseudomonas aeruginosa biofilms using ATR-IR spectroscopy. Langmuir.

[pgen-0030053-b054] Chang W, Hsu G, Chiang S, Su M (2006). Heavy metal removal from aqueous solution by wasted biomass from a combined AS-biofilm process. Bioresour Technol.

[pgen-0030053-b055] Ewing B, Hillier L, Wendl MC, Green P (1998). Base-calling of automated sequencer traces using phred. I. Accuracy assessment. Genome Res.

[pgen-0030053-b056] Ewing B, Green P (1998). Base-calling of automated sequencer traces using phred. II. Error probabilities. Genome Res.

[pgen-0030053-b057] Gordon D, Abajian C, Green P (1998). Consed: a graphical tool for sequence finishing. Genome Res.

[pgen-0030053-b058] Bocs S, Cruveiller S, Vallenet D, Nuel G, Médigue C (2003). AMIGene: Annotation of MIcrobial Genes. Nucleic Acids Res.

[pgen-0030053-b059] Claudel-Renard C, Chevalet C, Faraut T, Kahn D (2003). Enzyme-specific profiles for genome annotation: PRIAM. Nucleic Acids Res.

[pgen-0030053-b060] Krogh A, Larsson B, von Heijne G, Sonnhammer ELL (2001). Predicting transmembrane protein topology with a hidden markov model: Application to complete genomes. J Mol Biol.

[pgen-0030053-b061] Bendtsen DJ, Nielsen H, von Heijne G, Brunak S (2004). Improved prediction of signal peptides: SignalP 3.0. J Mol Biol.

[pgen-0030053-b062] Lowe TM, Eddy SR (1997). tRNAscan-SE: A program for improved detection of transfer RNA genes in genomic sequence. Nucleic Acids Res.

[pgen-0030053-b063] Vallenet D, Labarre L, Rouy Z, Barbe V, Bocs S (2006). MaGe: A microbial genome annotation system supported by synteny results. Nucleic Acids Res.

[pgen-0030053-b064] Tang X, Lu BF, Pan SQ (1999). A bifunctional transposon mini-Tn5gfp-km which can be used to select for promoter fusions and report gene expression levels in Agrobacterium tumefaciens. FEMS Microbiol Lett.

[pgen-0030053-b065] Carapito C, Muller D, Turlin E, Koechler S, Danchin A (2006). Identification of genes and proteins involved in the pleiotropic response to arsenic stress in Caenibacter arsenoxydans, a metalloresistant beta-proteobacterium with an unsequenced genome. Biochimie.

[pgen-0030053-b066] Hommais F, Krin E, Laurent-Winter C, Soutourina O, Malpertuy A (2001). Large-scale monitoring of pleiotropic regulation of gene expression by the prokaryotic nucleoid-associated protein, H-NS. Mol Microbiol.

[pgen-0030053-b067] Médigue C, Krin E, Pascal G, Barbe V, Bernsel A (2005). Coping with cold: The genome of the versatile marine Antarctica bacterium Pseudoalteromonas haloplanktis TAC125. Genome Research.

[pgen-0030053-b068] Kayser MF, Stumpp MT, Vuilleumier S (2000). DNA polymerase I is essential for growth of Methylobacterium dichloromethanicum DM4 with dichloromethane. J Bacteriol.

[pgen-0030053-b069] Parales RE, Ditty JL, Harwood CS (2000). Toluene-degrading bacteria are chemotactic towards the environmental pollutants benzene, toluene and trichloroethylene. Appl Environ Microbiol.

[pgen-0030053-b070] Shevchenko A, Sunyaev S, Loboda A, Shevchenko A, Bork P (2001). Charting the proteomes of organisms with unsequenced genomes by MALDI-quadrupole time-of-flight mass spectrometry and BLAST homology searching. Anal Chem.

