# Supplementary material for: A Tale of Two Oxidation States: Bacterial Colonization of Arsenic-Rich Environments
Source: PLoS Genet. 2007 Apr 13;3(4):e53. doi: 10.1371/journal.pgen.0030053 (PMC1851979; doi:10.1371/journal.pgen.0030053)
Supplement: Supplementary file 1 [file pgen.0030053.st001.doc]

**Table S1.** Insertion sequences identified in the *Herminiimonas arsenicoxydans* genome

Only ISs with at least one complete copy in the genome are named **(**[http://www-is.biotoul.fr](http://www-is.biotoul.fr/)).

| IS family | IS subgroup | IS name | Full copies | Partial copies |
| --- | --- | --- | --- | --- |
| IS*3* | IS*150* | IS*Har1* | 2 | 1 |
|  | IS*3* | IS*Har2* | 5 |  |
|  | IS*51* | IS*Har7* | 2 | 1 |
|  | IS*407* | - |  | 1 |
| IS*30* |  | IS*Har4* | 1 | 1 |
|  |  | IS*Har5* | 1 |  |
| IS*110* | IS*1111* | IS*Har3* | 1 | 1 |
|  |  | IS*Har6* | 1 |  |
| IS*5* | IS*427* | - |  | 1 |
| IS*21* |  | - |  | 3 |
| Tn*3* |  | - |  | 1 |
