# Supplementary material for: A Tale of Two Oxidation States: Bacterial Colonization of Arsenic-Rich Environments
Source: PLoS Genet. 2007 Apr 13;3(4):e53. doi: 10.1371/journal.pgen.0030053 (PMC1851979; doi:10.1371/journal.pgen.0030053)
Supplement: Supplementary file 2 [file pgen.0030053.st003.doc]

**Table S3.** Carbon and energy metabolism in *Herminiimonas arsenicoxydans*

| **Metabolism** | **Function** | **Gene identifier** |
| --- | --- | --- |
| Lactate | Lactate permease | HEAR2485 |
|  | L-lactate dehydrogenase | HEAR1710 |
|  | D-lactate dehydrogenase | HEAR2733 |
| Acetate | Acetate kinase | HEAR1515 |
| Succinate | Succinate dehydrogenase (Complex II) | HEAR1775-1778 |
| Fumarate | Fumarate reductase | HEAR3327-3329, HEAR3333 |
| Formaldehyde | Glutathione dependent formaldehyde dehydrogenase | HEAR2039 |
|  | S-formylglutathione hydrolase | HEAR2036 |
|  | Formaldehyde dehydrogenase | HEAR2048 |
| Formate | Formate dehydrogenase FdhN | HEAR1412-1413, HEAR1415-1418 |
|  | Formate dehydrogenase-NAD dependent | HEAR3430-3435, HEAR0356-0358, HEAR3442 |
| Aerobic respiration | *caa*_3_ cytochrome oxidase I | HEAR1115-1119 |
|  | *caa*_3_ cytochrome oxidase II | HEAR2915, HEAR2918-2919 |
|  | *bo*_3_ quinol oxidase | HEAR2699-2702 |
|  | *cbb*_3_ cytochrome oxidase | HEAR1641-1644 |
|  | *bd* quinol oxidase | HEAR1372-1375 |
|  | NADH-dehydrogenase (Complex I) | HEAR1813-1826 |
|  | *bc*_1_ ubiquinol oxidoreductase (Complex III) | HEAR3050-3052 |
| Sulfur oxidation | Sulfur-sulfite dehydrogenase | HEAR1188-1189 |
|  | Thiosulfate binding | HEAR1191-1192 |
|  | Cytochrome *c* | HEAR1193-1194 |
|  | Sulfur thiol esterase | HEAR1195 |
|  | Sulfite:cytochrome *c*  oxidoreductase | HEAR2348-2349 |
| Sulfite oxidation | APS reductase | HEAR2394 |
|  | ATP sulfurylase | HEAR2395-2396 |
|  | Sulfite reductase | HEAR2392, HEAR3398 |
| Denitrification | Nitrate reductase | HEAR1659-1662 |
|  | Nitrite reductase | HEAR3245 |
|  | Nitrous oxide reductase | HEAR3248 |
|  | Nitric oxide reductase | HEAR0048 |
| Arsenite oxidation | Arsenite oxidase | HEAR0478-0479 |
|  | Cytochrome c552 | HEAR0476 |
| Poly-beta-hydroxybutyrate | ß-ketothiolase | HEAR0577 |
|  | Acetoacetyl-coenzyme A reductase | HEAR0578, HEAR2345 |
|  | Poly-beta-hydroxybutyrate polymerase | HEAR1923, HEAR2340 |
|  | Poly-hydroxybutyrate depolymerase | HEAR0801 |
| Phophorus uptake | Inorganic phosphate transport (Pit) | HEAR2156 |
|  | Phosphate-specific transport (Pst) | HEAR486-491, HEAR1107-1111 |
| Phosphite oxidation | Phosphite transporter | HEAR3211-3213 |
|  | Phosphite oxidase | HEAR3214 |
| Phosphate storage | Polyphosphate kinase | HEAR1106 |
|  | Pyrophosphatase | HEAR1011 |
|  | Pyrophosphate-energized proton pump | HEAR2486 |
