# Supplementary material for: A Tale of Two Oxidation States: Bacterial Colonization of Arsenic-Rich Environments
Source: PLoS Genet. 2007 Apr 13;3(4):e53. doi: 10.1371/journal.pgen.0030053 (PMC1851979; doi:10.1371/journal.pgen.0030053)
Supplement: Supplementary file 3 [file pgen.0030053.st004.doc]

**Table S4.** Minimal inhibitory concentration (MIC) of toxic ions in *Herminiimonas arsenicoxydans* ULPAs1, *H. fonticola* S94 and *Ralstonia metallidurans* CH34

| **Metal** | **Strain** | **mM** | **ppm** |
| --- | --- | --- | --- |
| As[III] | ULPAs1 | 5,00 | 400 |
|  | CH34 | 1,25 | 100 |
|  | S94 * | 0,625 | 50 |
| As[V] | ULPAs1 | 100 | 8000 |
|  | CH34 | 50 | 4000 |
|  | S94 | 50 | 4000 |
| Cd^2+^ | ULPAs1 | 0,625 | 70 |
|  | CH34 | >10 | >1000 |
|  | S94 | 0,02 | 2 |
| Co^2+^ | ULPAs1 | 0,31 | 20 |
|  | CH34 | 10 | 600 |
|  | S94 | 0,16 | 10 |
| Cu^2+^ | ULPAs1 | 0,08 | 5 |
|  | CH34 | 1,250 | 80 |
|  | S94 | 0,16 | 10 |
| Cr[III] | ULPAs1 | 10 | 520 |
|  | CH34 | 10 | 520 |
|  | S94 | 5 | 260 |
| Zn^2+^ | ULPAs1 | 0,31 | 20 |
|  | CH34 | 5,00 | 300 |
|  | S94 | 0,08 | 5 |

** H. fonticola* S94, which has been isolated from a non-contaminated environment, was used as a control
